# Priority pathogens and the antibiotic pipeline: an update

**DOI:** 10.2471/BLT.20.251751

**Published:** 2020-03-01

**Authors:** Peter Beyer, Sarah Paulin

**Affiliations:** a Department of Global Coordination and Partnership, World Health Organization, avenue Appia 20, 1211 Geneva 27, Switzerland.

The steady rise of antimicrobial resistance is one challenge where the current research and development system does not provide the needed solutions. Two new World Health Organization (WHO) reports show that too few new antibacterial treatments are in development and that more interventions are needed.

The 2019 WHO clinical antibacterial pipeline analysis describes all antibiotics and biological treatments that are currently in development against the WHO priority pathogens list.^1^ The outlook is bleak: only 60 products are in the clinical phases 1 to 3.^1^ Of these antibacterial agents, 32 antibiotics are active against the WHO priority pathogens, 12 against tuberculosis and six against *Clostridioides difficile*. The 10 biological treatments in clinical development target *Staphylococcus aureus* (six treatments), *Pseudomonas aeruginosa* (two treatments) and *Clostridioides difficile* (two treatments).

WHO also assessed the novelty of the antibacterials using four criteria: absence of known cross resistance, new class, new target and new mode of action as defined by the WHO expert group. Only six of the agents that target the WHO priority pathogens fulfil at least one of the four criteria.

New derivatives of the same class can be superior than the first in class treatment by having a better safety profile, better efficacy or activity against resistant bacteria. However, resistance is likely to develop quicker against derivatives of the same class that share the same mode of action and target. Clinicians are reluctant to switch to new, more expensive treatments that are based on non-inferiority trials that only show that the new treatments are not worse than the standard of care. This reluctance and the conservative approach required towards using new antibiotics under stewardship programmes is translating into serious economic challenges. The recent bankruptcy of some of the small antibiotic research and development companies and the fact that most major pharmaceutical companies have left the antibiotic research and development space illustrates these economic difficulties.^2^

How to tackle the lack of private investment in the research and development of new antibacterial treatments has been discussed in the G7, G20 and other international fora.^3^^,^^4^ Driven by a few governments and organizations, some successful initiatives that provide push funding for antibiotic development and access to new antibacterial treatments have been set up. For example, initiatives of the Biomedical Advanced Research and Development Authority, the public–private partnership CARBx (Combating Antibiotic-Resistant Bacteria Biopharmaceutical Accelerator) and the Global Antibiotic Research and Development Partnership. The impact of these initiatives can already be seen in the preclinical pipeline.

WHO’s first report and publicly accessible database on the preclinical antibacterial pipeline published in January 2020 captures 252 antibacterial agents being developed by 145 individual institutions against the WHO priority pathogens, *Mycobacterium tuberculosis* and *Clostridioides difficile*.^5^ Overall, the preclinical pipeline is dynamic and scientifically diverse with over one third of the projects focused on non-traditional projects. However, most of these projects are likely to fail with only a handful making it to the market given the enormous scientific challenges for some of these non-traditional approaches^6^ that are not yet proven to work in a clinical environment, and the lack of well-defined regulatory pathways.

More public investment is needed to ensure a viable economic environment for antibacterial treatments that are innovative and add significant clinical value. Major pharmaceutical companies also have to make a more sustainable financial contribution. Antibiotic developers together with regulatory agencies must find ways to better demonstrate the clinical advantages of new antibiotics over standard of care through clinical data. Maintaining the antibiotic research and development crisis high on the international political agenda is essential to push for further reforms and to ensure that bacterial infections do not become another field of neglected diseases.

In line with WHO’s mandate to promote and conduct research in the field of health,^7^ the organization will continue to track the antibacterial preclinical and clinical development pipeline, expanding to non-traditional products, bacterial vaccines and antifungals. WHO will also develop target product profiles for missing products and support the Global Antibiotic Research and Development Partnership as an independent global research and development entity, as well as other research and development initiatives, to ensure that these efforts focus on public health needs.
